# ASSESSMENT OF PALLIATIVE CARE TRAINING NEEDS FOR CERTIFIED NURSING ASSISTANTS

**DOI:** 10.1093/geroni/igz038.1087

**Published:** 2019-11-08

**Authors:** Jinsook Kim, Jennifer A Gray

**Affiliations:** Northern Illinois University, DeKalb, Illinois, United States

## Abstract

It is unknown whether certified nursing assistants (CNAs) receive up-to-date palliative care training through continuing education. Also unclear is whether existing trainings cover the issues that CNAs encounter at work or are tailored to CNAs’ learning styles and preferences. This study aimed to assess the palliative care training needs of CNAs working at skilled nursing facilities (SNFs) in northern Illinois. CNAs (n=127) from 6 SNFs completed an online survey regarding palliative care training experience, perceived needs for palliative care training, and demographic and work-related information. The majority of the participants were female (88%) and White (58%) or African American (20%). On average, participants were 34 years old and worked for 8 years in the field. Four out of five preferred a training 90 minutes or shorter. Approximately one half preferred in-person training, and the rest preferred a hybrid (32%) or online delivery (19%). Discussions and videos were most preferred in training, while quizzes and mobile apps were least preferred. CNAs who worked longer in the field were less likely to have received training than their counterparts. The least-trained areas included utilizing advance directives and discussing death with patients. The most needed training areas were talking to a patient who wants to hasten death and addressing the complex needs of dying patients. The results indicate a relative deficiency of palliative care training among CNAs who have worked longer in the field. Training areas needing more attention include advance directives, discussing death with patients, and the complex needs of dying patients.

